# Youth Impacted by a Family Member’s Substance Use: A Qualitative Exploration of Life Course Experiences and Coping

**DOI:** 10.1007/s40653-026-00869-4

**Published:** 2026-04-10

**Authors:** Cathrine Hørte, John-Kåre Vederhus, Eirik Abildsnes

**Affiliations:** 1 Addiction Unit, Sørlandet Hospital Kristiansand, Kristiansand, Norway; 2 University of Agder, Grimstad, Norway; 3 University of Oslo, Oslo, Norway

**Keywords:** Adverse childhood experience, Substance-related disorders, Family, Coping skills, Qualitative, Sense of coherence

## Abstract

Many reports describe how children and youth are negatively impacted by substance use in their household. Less is known about how they cope. Moreover, there is a scarcity of research that analyses empirical data through a theoretical lens. The aim of the present study was two-fold: to explore the experiences and coping strategies of youth growing up impacted by a family member’s substance use, and to examine how these findings relate to pertinent theory. The participants were 17–21 years of age and had participated in a psychoeducational programme for affected family members. We conducted in-depth interviews (*n* = 6), applying Systematic Text Condensation in a thematic cross-case analysis. Discussions of the findings were informed by insights from the Salutogenic Model of Health. Two main themes emerged. First, growing up with substance use is a lonesome and demanding experience. Second, children and youth cope using methods that may become problematic—such as supressing emotions and trying to be self-supported—as well as strategies aimed at fostering independence and focusing on constructing a better future. By discussing our findings in the context of salutogenic theory, we gained insights into the multifaceted aspects of coping, which may inform practice and facilitate movement towards health in this population.

## Background

A safe and nurturing upbringing from childhood and through adolescence increases the likelihood of a healthy and well-functioning life in adulthood. Conversely, exposure to adverse childhood experiences (ACE) is associated with increased vulnerability to illness and difficulties in social and economic functioning later in life. The original ACE study found that substance use in the household was among the most prevalent categories of childhood exposures to abuse and household dysfunction, reported by 25% (Felitti et al., 1998). Globally, 435 million of the adult population has a substance use disorder (SUD), affecting the lives of many children and youth (UNODC, 2024; World Health Organization, 2024). In Scandinavia, it is estimated that 5–8% of children live with a parent having a SUD (Raninen et al., 2016; Torvik & Rognmo, 2011). The actual proportion affected is likely to exceed the reported prevalence of diagnosed SUDs within households, particularly when accounting for variations in usage patterns, severity and the potential for underreporting (Kraus et al., 2021).

Previous studies have explored how substance use puts considerable strain on family members (Orford et al., 2010). Lindeman et al. (2022) portrays the presence of substance use in a family as “an intrusion that overshadows all aspects of life”. The impact is often invisible to outsiders and is often perceived as a family secret. The family becomes locked into demanding situations and must continuously adapt. While acknowledging considerable strain to partners, children and youth are the most vulnerable, as they cannot escape and often take on caregiving responsibilities due to reversal of roles (Lindeman et al., 2022; Weimand et al., 2020). Over recent decades, the existing knowledge has expanded regarding the lifetime burden and consequences of adversities in childhood, including substance use in the household (Felitti, 2019; Hughes et al., 2017). Prolonged exposure to childhood adversities can have toxic effects on children leading to physical and psychosocial impairments and increases the likelihood of mood disorders, risky health behaviour, and behavioural problems (Hughes et al., 2017).

Considering the possible negative impacts on the health of the affected individual in relation to their ways of coping with adversities, it becomes relevant to apply the Salutogenic Model of Health (SMH) and more specifically its core concept Sense of Coherence (SOC) as a theoretic framework (Antonovsky, 1979, p. vii). The SOC is conceptualized as comprising the three interrelated dimensions comprehensibility, meaningfulness and manageability – which encapsulate the cognitive, motivational and behavioural processes involved in the interpretation and reaction towards environmental stimuli (Mittelmark & Bauer, 2022). SOC is a flexible coping resource. In this model, even if tension and stress management may be detrimental to physical and mental well-being, it may also initiate processes that enhance overall health and coping capacity (Vinje et al., 2022). SOC coexists with generalized resistance resources, i.e., the resources that facilitate one’s ability to cope, which are formed by cultural, social, and environmental conditions, and may exist at the individual, family, and/or community level (Mittelmark et al., 2022; Orly et al., 2022). SOC and general resistance resources exhibit a dynamic relationship and may reciprocally contribute to each other—with general resistance resources strengthening SOC, and SOC enabling mobilisation and use of resistance resources. Thus, SOC contributes to effective coping in the face of stress and adversities (Orly et al., 2022).

Review of qualitative studies has showed that much of the research in this field has focused on how substance use in the household affects children and youth—with emphasis on dysfunctional coping strategies and negative outcomes, and less recognition and analysis of their efforts to cope (Muir et al., 2022). Our aim was to explore life course experiences of youth impacted by a family member’s substance use, and their reflections on what they did to cope. Few studies have qualitatively examined this group of youth’s experiences within a salutogenic framework (Bauer, 2017; Shorter et al., 2023). Intending to balance the descriptions of adversities and capabilities, we employed the concept of SOC and resistance resources to guide the interpretation while discussing our findings.

## Method

Here we explored lived experiences using qualitative methodology, including methods founded in hermeneutic phenomenology, which focuses on the human experience as it is lived (Malterud, 2012).

### Participants

We recruited participants with purposeful sampling, enabling us to include participants who could describe their own experiences as experts, answering to the research aim (Lefèvre et al., 2019). The participants attended a 4-day psychoeducational program that was offered to close relatives of patients with SUD, at the Addiction Unit of NN. Apart from prior participation in the psychoeducational program, no additional inclusion or exclusion criteria were established before recruitment. In cooperation with a psychoeducational program staff member, the first author contacted 20 persons who attended the program. Six of the contacted persons agreed to participate in our present study, each of whom had been involved in the program within the last 24 months, and all had a recent memory and an ongoing experience of living with a family member with SUD. Participant suitability for inclusion was evaluated collaboratively by the psychoeducational staff and the first author. There was no severe psychiatric comorbidity, and all participants had contact with the substance using family member.

Our sample (Table 1) included two men and four women in the age range of 17–21 years, referred to as ‘youth’, following the UN’s statistical definition of this age group, all from different families (United Nations, n.d.). All participants had experienced substance use within their families from toddlerhood or early childhood, with varying relationships to the family member with SUD and differences in substance involved. Four participants had divorced parents and two had siblings with SUD. The participants’ presentations represented both ongoing experiences and retrospective insights.

**Table 1:** In-depth interview participant characteristics

In-depth interviews	Sex	Age	Family member with SUD	Substance (s) used by family member
1 “Eric”	Male	21	Father & brother	Alcohol & Alcohol and drugs
2 “Anna”	Female	18	Father	Alcohol and drugs
3 “Roy”	Male	20	Mother	Alcohol and drugs
4 “Sara”	Female	18	Father	Alcohol
5 “Lisa”	Female	17	Brothers	Alcohol and drugs
6 “Tina”	Female	20	Mother	Prescribed drugs

*SUD *= Substance Use Disorder

## Data Collection

We developed a semi-structured interview guide in collaboration with a user participant to ensure that the questions were ethically sensitive and meaningful for this group of youth (Schweiger, 2024). The guide incorporated exploratory questions related to the scope of this study, which included the participants’ experiences having a family member with a SUD, and the participant’s coping needs. The first author conducted the individual interviews. On one occasion, the last author participated as an observer for quality assurance. Interviews were conducted in 2018, lasted from 45 to 65 min, and took place in the researcher’s office or at a location of the participant’s choice, respecting their confidentiality. The interviews were audiotaped, and transcribed verbatim by the first author, partly in cooperation with a research assistant.

### Data Analysis

For our analysis we applied Systematic Text Condensation (STC), which is a suitable method for thematic cross-case analysis of various types of qualitative data (Malterud, 2012). This pragmatic approach elaborates on Giorgi’s phenomenological method and principles. It is a descriptive approach that aims to present people’s life experiences, rather than all potential phenomena. STC represents an editing analysis style with an inductive approach (Crabtree & Miller, 2022, p. 22). The analysis contains four analytical steps. First, we read through all transcripts to get an overall impression and identify preliminary themes, consciously striving to bracket our own preunderstandings, by actively discussing them. Subsequently, themes were critically evaluated and prioritized for further analysis. Second, we identified and sorted meaning units related to the previously named themes (code groups)—a process called de-contextualization. In the third step, condensation, we systematically abstracted meaning units in the established 3 code groups into 9 subgroups, and the content was reduced to a “condensate”—an artificial quotation maintaining the original terminology used by the participants. Although no contradictory cases emerged, nuanced variations in participants’ experiences were evident and captured through subgroup themes. Finally, we synthesized and re-conceptualized the data. The contents of the condensates and quotations from each subgroup were synthesized and developed as an analytical text. Applying an inductive approach, we discussed the choice of theories at the condensation stage with all data at hand. The discussion of findings was informed by insights from the SMH and SOC (Varpio et al., 2020). To validate the findings, we re-contextualized the analytic texts against the full transcripts, reflecting on concepts and expressions, and verifying that our understanding accurately represented the transcripts. To complete the analysis, data were compared to existing knowledge. The user participant provided feedback on the results, confirming that the results reflected recognizable experiences of PSU within the family, thereby validating the context of the presenting themes. The research team engaged in reflexive practices regarding how our roles and backgrounds might influence the research process. A fuller reflexive account is presented in the Discussion section.

### Information Power

The aim of this study was narrow in scope: to examine youth who had attended a psychoeducational program for family members affected by a close relative’s SUD, exploring experiences of how the family member’s substance use affected their lives and the coping needs this elicited. The sample specificity was high for the study’s purpose, including youth who all had experiences growing up with a family member with a severity of substance use eventually qualifying for a SUD. Additionally, despite the relatively small sample size, the sample held diversity regarding variations to whom the participants were related; each kind of parents, a sibling or both, and as to which type of substance was involved, contributing to the richness and relevance of data. The interviewer possessed competency and skills to achieve a good dialogue with the participants. We performed a thematic cross-case analysis to obtain descriptions of the various experiences and applied a theoretical framework and concept, SMH / SOC, explaining relations between aspects seeking to strengthen the information power. Assessing these items and considering the sample size, we judged the overall information power to be sufficient to conduct the analysis (Malterud et al., 2016).

### Ethical Considerations

We followed the principles of the Helsinki Declaration, conducting the study as approved by The Regional Committee for Medical and Health Research Ethics (No 2015/1561) (World Medical Association, 2013). When the participants were recruited by telephone, they received information about the study and confidentiality and gave their preliminary oral consent. On the day of interview, participants received detailed written information about the research project, its procedure, and confidentiality measures prior to signing an informed consent. We made every effort to apply our clinical expertise during the interviews to identify any signs of emotional distress. Participants were asked how they were doing and offered breaks as needed. We offered the possibility of referral to a therapist if participants experienced any negative emotional consequences. We did not encounter any instances where participants disclosed information that required mandatory reporting. When presenting the results, identifiable details are removed, and participants are given pseudonyms when referring to their quotes.

## Findings

**Fig. 1:**
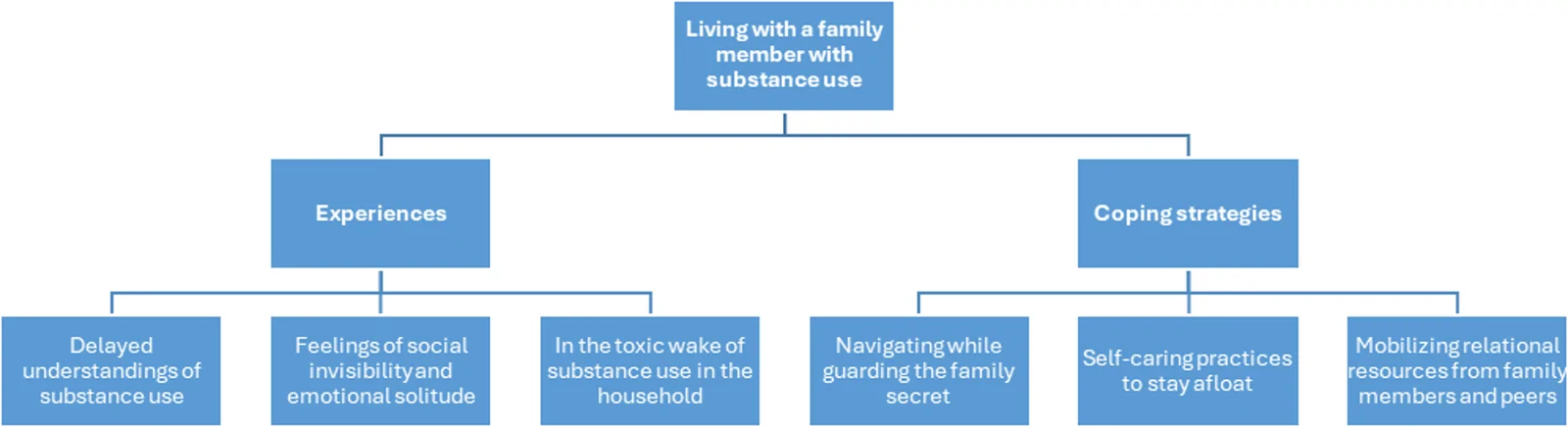
Thematic map outlining the two overarching themes—experiences and coping strategies—related to living with a family member with substance use, each accompanied by three subthemes that further elaborate their content

The results are organized and presented based on the aim of the research —with three themes focusing on the youths’ experiences of growing up having a family member with substance use and three themes exploring their coping strategies, Fig. 1. These findings are presented descriptively below and will be analysed through a salutogenic lens in the subsequent section.

### Experiences

#### Delayed Understandings of Substance Use

Participants had experienced substance use in their families from infancy. Even in early childhood, they felt that something was wrong. They sensed that their family differed from others but could not identify the problem. In hindsight, they recognized that, during their preschool years, the people around them had tried to shield them from reality by withholding information or lying about what was going on for a long time. Anna (aged 16) explained, “My father did drugs. …when I was thirteen … I was old enough to understand what was happening, sort of, and my mum couldn’t hide it anymore”.

Some participants that lived part time with each parent suggested that this concealed the substance use to some extent and rendered the participant more alone in the situation. Others first became aware that substance use was the problem in late childhood and early youth. Roy (aged 18) described observing as a teenager that his mother only had friends who used drugs. Tina (aged 20) said that her mother had a somatic pain condition that initiated her dependency on painkillers, a process that was difficult to understand: “I noticed that my mum … well I did not know really what it was. I cannot remember how she was when she was sober, sorry to say. I didn’t really know anything else.” Some participants did not fully realize that their parent or sibling was addicted to substances until late teens, when their relative accepted treatment.

#### Feelings of Social Invisibility and Emotional Solitude

Several participants specified feelings of being invisible, not receiving attention from adults, and growing up not understanding what was going on. This feeling depended on their emotional relationship with the parent who did not use substances. Some participants experienced that this parent maintained an emotional distance, and had difficulties expressing feelings, which contributed to the struggle of making sense of it all.

Participants perceived their family as different from their peers’ families. Some expressed a sense of reluctance to bring friends to their homes due to fear of what might ensue. Tina (aged 20) experienced feelings of confusion, loneliness, and insecurity, saying: “If we had told about it, we might not have been taken seriously. I never talked to anyone about it, maybe because of fear … maybe there isn’t anything wrong?”. The participants described friends’ and family members’ insecurities and shortcomings in responding to the attempts to involve them in the household situation. Some participants excused the lack of understanding, explaining that their challenges were difficult to detect. Others found it peculiar and disappointing when adults in the family, or amongst friends and neighbours, did not act, such as Sara (aged 17):

My aunt, she knew … I don’t think she knew what to do, but she didn’t even talk to mum or us about it … I wish she would’ve asked us how we were doing or if we noticed that something wasn’t right. … Our neighbours saw a lot but said nothing. Later, they’ve said that they were thinking of contacting the child protective services, but they did nothing.

#### In the Toxic Wake of Substance Use in the Household

Participants gave multifaceted descriptions of the burdens of living close to a relative with substance use. Some shared memories from scenarios of negotiation, rage, and violence by the substance user, which left them in a worried and unsafe state. Anna (aged 18) described nights overwhelmed by tears and haunted by nightmares. Others described fluctuating symptoms of parental depression and apathy. Tina (aged 20) said that her mother distanced herself from family life, causing worry that her parents would divorce. Several participants described shame, giving examples of when their substance-using parent crashed the car, or was overtly influenced by substances in public. Participants often experienced the need to assume a parental role in the family, managing daily chores that are usually handled by parents. Some described a lack of rules and curfews and being left to manage by themselves. For Eric (aged 21), the total burden became overwhelming:

Eric: Well, the first time I wanted to hang myself, I was 10–11 years old … but my mum came in the door at home. Interviewer: Did she find you as you planned for it? Eric: No, I heard her enter the door, so I put down the rope. Interviewer: Was this in a direct connection to your home conditions? Eric: Yes, it was because I was so exhausted.

Several participants described a childhood coloured by insecurity and self-anger over letting the troubles in on them; they did not want to bother others with their worries. Participants emphasized feeling powerless and desperate due to not being able to help their relative, who often denied substance use and refused treatment. Participants worried for their relative’s well-being, such as fearing a sibling’s overdose. Others described the need to constantly check on their parent. Some felt they were part of a play that they didn’t understand. Tina (aged 20) told of how her mother used her and her siblings to buy prescription drugs on the black market.

At school age, participants often regarded their struggles at home as more demanding than school itself. Some characterized attending school as “time off”. Tina (aged 20) felt that school was her “happy space”, where she could leave her troubles at home. Several participants described how over time, their burdens caused them to fail at school, due to not being able to mentally focus and having absences. Tina stated that her long-standing difficult home conditions eventually caused severe migraines at school. The described strains resulted in dropping grades and the need for a longer break from school, accompanied by low self-esteem and feelings of depression, shame, and isolation.

### Coping Strategies

#### Navigating While Guarding the Family Secret

Participants displayed a variety of methods of coping with a family member using substances. Several participants had intuitively kept their home conditions a family secret, describing how they concealed their hardships by avoiding talking about their problem and suppressing their emotions. They described how they locked up inside, isolating themselves and keeping friends at a distance. Anna (aged 18) explained, “I was a lot like, wanting to be left alone …I withdrew from my friends … I didn’t want to open up for it … I didn’t want them to show concern”. Some disclosed their concerns to more distant family members who knew about the circumstances. Tina (aged 20) never talked to anyone at all about her mother’s substance use until her mother commenced treatment. While Eric (21) reflected on his own way of coping:

I didn’t even talk to my siblings about it. I didn’t ask them if they also noticed that something was wrong … I think I kept it a lot to myself … it might not have been the best option, but that’s how I did it.

Similarly, others hid their struggles and did not want to receive help. Roy (aged 18) said he believed that his family situation only affected him at a subconscious level, despite having several close family members using substances. Participants avoided talking about their situation to prevent separation from their family. Tina (aged 20) said she withdrew from her friends because she did not want to be a burden to them. Some said they strived to be and act like their peers, coping on their own. Eric (aged 20) said that pride prevented him from opening up. Other participants kept their thoughts to themselves, explaining that their friends lacked the background needed to acknowledge and understand because they had no similar experiences.

#### Self-caring practices to stay afloat

The participants characterized their everyday life as struggling with fluctuating emotions and mental well-being but also said that not all days were sad and difficult. Moreover, they described their inner notion that they had to do something for themselves to feel better. Eric (aged 20) said he practiced his own sort of mindfulness. Several wrote what they were going through in a diary, and some expressed their feelings by writing poems and songs. Anna (aged 18) said:

It helped me to discover what I could do to make myself feel better… if I couldn’t sleep at night, I wrote down my feelings and I felt better afterwards. One needs to find a way to express your feelings. … You know, it wasn’t as if you were in the pit every day, it was more like a roller coaster … and, when you are down you need something that pulls you up again.

Some mentioned the value of participating in leisure-time physical activities, which gave them a break where they had no worries and could enjoy their time and just be themselves. They also noted that physical exercise positively impacted their mood, such as Anna (aged 18) saying:

Friends and physical exercise were important to me, having something to go to, sort of … there, they didn’t mind what you were going through or how you felt. It was freedom training there, everything just disappeared, the only focus was cheerleading.

Once the participants recognized that their relative suffered from a SUD that impacted their personality, some found that it became easier to accept their situation and enabled them to limit their own involvement to protect themselves. This subsequently became a tool to handle disappointments.

Despite considerable burdens early in life, the participants commonly expressed optimistic views and hopes for the future. They chose to focus ahead, realizing that preoccupation with the past would lead to a depressive state. Erik (aged 21) was able to appreciate his hardships, saying:

For my part, I am quite thankful of what I have gone through as well, because I wouldn’t have developed in relation to others in the same way if not. I have matured and become more confident of who I am.

#### Mobilizing relational resources from family members and peers

Several participants described their parent who did not use substances as being caring and attentive and recognizing their needs. They opened up to this parent, being honest about their challenges and feelings troubling them. Participants emphasized that private conversations were valuable for making sense of what was going on in the family. In hindsight, they could see that the sober parent often strived to spare them of difficulties, and to help them understand the nature of substance use. Erik (aged 21) felt that his mother’s talks about mental health and reactions made him better equipped to cope with life. He further described how mutually important it was to him to know how his mother was coping, and to be able to also care for her.

Some participants emphasized the importance of learning from their sober parent how to say no, where to draw the line, and where to take their position in the family. Others were close to their siblings and could also confide in other family members (e.g., cousins) because of their acquaintance with the family situation.

Participants also highlighted the importance of friends. Lisa (aged 18) compared friendship to the relationship with a parent:

I talk to mum in the sense that he’s my brother [a brother who’s struggling with substance use] and her son. I talk to my friend because she is also a next-to-kin, experiencing similar challenges. She is my friend, it is something different having a friend, not just mum. I laugh with my friends … she’s helped me a lot, definitely.

The participants did not disclose sensitive information to random friends, rather the contrary. Anna (aged 18) said she used to test her friends to determine if they also had fathers who used alcohol. Trust was fundamental, and the participants more easily opened up to friends who shared a lived experience with a family member who used substances. Roy (aged 18) explained why: “People you talk to don’t always share your experience; they’ve only heard about it. When I talk to friends who have gone through similar or exactly the same, we get a whole lot more understanding.” Common experiences were discussed freely and sometimes with gallows humour. Participants perceived such friendships as unconditional. Some sought refuge at friends’ houses during the most challenging periods. Participants described a stepwise process of learning that sharing their troubles alleviated the pain and helped them cope.

## Discussion

We aimed to explore youths’ experiences growing up with substance use in their household, along with their methods of coping, and to discuss our findings in the context of SMH and SOC. Growing up with substance use was described as difficult to understand, lonely and emotionally demanding, constraining the SOC. Participants coped with these burdens using methods that may become problematic as well as salutogenic strategies.

### Burdens of Growing Up with a Family Member with Substance Use

Notably, the participants reported spending a significant portion of their childhood not realizing that substance use was at the root of the problem. They described feeling like something was wrong from a young age; however, in early childhood, they were not yet able to grasp the core of the problem and make sense of their situation. Childhood development features the acquisition of skills, knowledge and competencies over time (Keenan et al., 2016, p. 5). Thus, an age-related limited ability to conceptualize substance use may partly explain their experience of not being able to discern what was going on. Understanding the complexities of substance use is difficult at any age, and especially so for a developing child (Wangensteen et al., 2020). This expressed uncertainty about their home circumstances suggests that the comprehensibility dimension of SOC, the degree to which one’s experiences is understandable (Moksnes, 2021), may have been compromised. Consequently, this may interfere with the development of overall strength of SOC which starts in early childhood and depends on the quality of social interactions, and close relationships (Idan et al., 2022; Vinje et al., 2022). For children and young navigating adversity, comprehensibility may be considered a foundational element in facilitating manageability and cultivating the capacity for coping with their challenges (Antonovsky, 1987, p. 22).

In hindsight, participants in our study felt excluded and deprived of the opportunity to develop an understanding, such as Anna saying her mother hid the problem and that only as she grew older, she began to comprehend it. Some expressed frustration towards the adults in their lives for inaction and withholding information and even perceived it as lying under the guise of protection. It is understandable that adults might have hesitated to discuss the problem openly, possibly due to concerns about the child’s ability to comprehend the situation. However, previous research suggests that secrecy is common in families affected by substance use, along with feelings of shame and deviating from societal norms (Lindeman et al., 2022; Wangensteen et al., 2020). Thus, withholding information may have stemmed from a desire to preserve family secrets, rather than solely concerns about the child’s understanding.

Meanwhile, the children themselves want to understand, and seek information and knowledge in their struggle to make sense of their situation (Wangensteen et al., 2020). However, they also recognize the lack of normality and the risks of disclosure, such as participants in this study fearing separation and experiencing shame (Backett-Milburn et al., 2008; Lindeman et al., 2022). This accompanying shame, as a social emotion, can impair a young person’s emotional development and their ability to connect socially (Arcidiacono et al., 2009; Mayer et al., 2019; Muir et al., 2022). Our findings align with previous research showing that children’s secrecy reflects needs for privacy related to shame and loyalty, as well as a desire to exert control over their circumstances (Holmila et al., 2011; Velleman & Templeton, 2016). In addition to signalling social and emotional strain related to secrecy, these experiences may reflect disrupted development of the meaningfulness dimension of SOC.

The participants’ life situations suggest that they were in a less favourable position to take part and shape their daily experiences, outcomes, and fate, challenging the basis of meaningfulness. Meaningfulness, understood as the perceptions that life makes sense emotionally and is worthwhile of investment, becomes compromised when children and young lack the opportunity to actively participate in shaping outcomes (Antonovsky, 1987, p. 18; Meier Magistretti, 2022). Their fear of family separation represents a profound threat to perceptions of meaningfulness as it erodes the motivational basis for mobilizing resources and engage with challenges. Consequently, both secrecy and fear may significantly constrain this pivotal dimension of SOC by diminishing its strength and thereby impairing children and youth’s overall coping ability (Antonovsky, 1987, p. 22; Sagy & Antonovsky, 2000).

### Coping Strategies

Navigating in this challenging landscape required participants’ careful considerations and cautious steps. Investing in keeping the family secret became a balancing act of necessity, causing some of our participants to withdraw and keep a distance from their friends. Others avoided talking about their troubles and some decided to manage on their own. Stuck with a family member struggling with substance use, children and youth often find themselves lacking viable means to break free from their difficult circumstances. Yet, they experience a coexisting desire to take care of and stay involved in their family member’s life (Lindeman et al., 2022). Coping in such complexity may foster inauspicious strategies, as shown here and in prior research; suppressing emotions and relying on oneself can become a “double-edged sword”—helpful and even necessary in the short term, but potentially harmful over time. (Backett-Milburn et al., 2008; Hill, 2015; Lindeman et al., 2022; Muir et al., 2022).

Facing challenges while growing up may allow general resistance resources to emerge and help shape SOC and coping abilities (Orly et al., 2022). However, the participants’ careful balance of family secrets may parallel the balance of general resistance resources or their deficits within the SMH. Some of our participants were less able to draw on social support as a resistance resource, leading to a painful period of silence marked by difficult emotions, fears, and isolation. These reactions may indicate constraints challenging the development of manageability dimension, which entails having resources and capabilities to meet demands (Antonovsky, 1987, pp. 17–18; Orly et al., 2022). If challenged, its consequences may influence the tension posed to the developing SOC, a psychosocial process essential for mental health (Eriksson, 2022; Orly et al., 2022).

The participants also expressed that not all days were sad and difficult, and some emphasized that humour was important to get by. Intuitively and over time, participants gained an inner notion that they had to do something to care for themselves to feel better. To this end, they wrote diaries, practiced their own sort of mindfulness, wrote songs, and played instruments. They also found that physical activities and sports allowed them time off from everything. Existing research supports our participants’ descriptions of using their own “survival kit” to face adversities. Self-caring strategies such as emotional expression and humour can be understood as adaptive responses that ease negative emotions and support psychological well-being (D’costa & Lavalekar, 2021; Kuiper, 2012).

Additionally, their engagement in physical activities, which was linked to positive mood changes, highlights the role of exercise as a mechanism for affect regulation. When performed in a social setting, physical activity can strengthen resilience and offer enduring mental health benefits (Easterlin et al., 2019; Hughes et al., 2018; Massey & Williams, 2020; Silvén Hagström & Forinder, 2022). Meanwhile, despite their constrained and vulnerable circumstances, some participants’ coping actions demonstrate resiliency. They managed these tensions by drawing on internal general resistance resources, such as knowledge of own helpful coping strategies. Additionally, they found helpful more specific resistance resources, such as joining sports (Mittelmark et al., 2022). These are features reflecting a strong SOC, being able to mobilize and use strategies that seem appropriate to deal with stress (Antonovsky, 1987, p. 138; Mittelmark et al., 2022).

Coping with their tensions, some participants carefully reached out for social support, defying the risk of disclosing their family secret. Some dared opening up to friends whom they found capable of receiving their story, or to close adult family members. One participant described small bedside talks about his brother’s substance use with his attentive mother, which increased his understanding (i.e. comprehensibility) of the situation. In this mutual caring and understanding relationship, he learned to withdraw and care for himself (i.e. manageability), rather than focusing all his attention on the family member with the substance use (Orford et al., 2010). This helped him make greater emotional sense (i.e. meaningfulness) of his situation and protect himself.

Previous research indicates that seeking social support outside the family carries risks. Children and young people typically confide in those with whom they have close relationships, and as they mature, their help-seeking broadens to include non-familial sources (Muir et al., 2022). While seeking support is central to their coping, a trusting relationship appears essential - illustrated by this participant’s strong reliance on a nurturing parent (Backett-Milburn et al., 2008; Holmila et al., 2011). Perceived supportive relationships, in the context of familial substance use, are characterized by care and compassion, which enhance trust and safety (Muir et al., 2022). This participant’s story can be interpreted as positioning the trusting relationship as a resistance resource within the coping process and suggests how the overall SOC may be strengthened within a confident social relation (Antonovsky, 1987, pp. 17–18). Understanding his situation increased both meaningfulness (by elevating his emotional sense) and manageability (by enabling him to withdraw). This may further indicate how social support, as a general resistance resource, interplays with and may strengthen SOC, thereby further enabling coping (Orly et al., 2022).

### Contributions - Experiences and Coping in Context of SMH and SOC

The SOC construct reflects a person’s capacity to cope with stressful situations. When examining our findings relative to the three dimensions of SOC, we gained insight into their interconnectedness, underscoring the multifaceted aspects and processes of growing up in households affected by substance use (Antonovsky et al., 2022). This provides insight into ways social circumstances may influence the strength of SOC (Joseph & Sagy, 2022). Our findings demonstrate how the stress of living with substance use may influence children and youth’s lives and constrain their capacity to cope across all dimensions. Yet our results also highlight coping skills and traces of resiliency along the way from childhood into youth, as cognitive development influences the motivational and behavioural dimensions of SOC (Braun-Lewensohn et al., 2022).

Complementing frameworks focused on protection and risk reduction, our study underscores SOC as a resilience and health promoting factor and elucidates how it may relate to the mobilisation of resistance resources when facing adversity (da-Silva-Domingues et al., 2022; Eriksson, 2022; Mittelmark & Bauer, 2022; NICE, (2009). Hence, our study enriches current knowledge by exploring how a qualitative enquiry, supported by salutogenic theory, may provide insight into social context and possible pathways to strengthening life resources and positive health development while amplifying the often-overlooked perspectives of this group of adolescents (Álvarez et al., 2021; Bauer, 2017; Bauer et al., 2020). The study can inform interventions and psychoeducational programs by exploring SOC dimensions and GRRs as an approach to understand the complex life situations of individuals affected by a family member’s substance use. This perspective emphasizes mapping potential resources as a constructive focus rather than merely highlighting shortcomings, deficiencies, and constraints. Such an approach may strengthen overall SOC and uncover external and internal resources that together support and mobilize beneficial coping (Langeland et al., 2022; Pérez-Wilson et al., 2021).

### Limitations

This study is subject to methodological limitations related to trustworthiness. Given the purposeful and pragmatic sampling of a limited number of former participants from a psychoeducational program for families affected by a member’s SUD, the transferability of these findings to other contexts should be approached with caution.

Considering the continued need for more qualitative insights to the experiences and needs of children and youth positioned growing up with a family member diagnosed with a condition qualifying for health services, such as SUD, the sample was found adequate for the study’s exploratory aims (Ahmad & Wilkins, 2025; Ytterhus et al., 2025). However, this study does not explore the extent to which experiences associated with sibling substance use may differ in nature from those involving parental substance use, as our focus here was on commonalities.

Throughout the interview process we verified that the participants possessed relevant experiential knowledge aligned with the research aim and provided detailed and rich descriptions of experiences while representing a variety in family relations and substance use involved (Malterud et al., 2016). However, we do acknowledge that few youths who have experienced substance use in the family are given the opportunity to participate in a psychoeducational program and that the specificity of the sample limits the transferability to the general population of children and youth living under such conditions. Furthermore, recall bias inherent in retrospective reporting may have constrained the study, as distressing experiences can be repressed and memories may weaken. Additionally, the potential for social desirability bias cannot be ruled out, as participants may have moderated their responses when addressing sensitive family experiences.

The participants’ involvement in the psychoeducational program might have influenced their rich and reflective narratives and should be considered when interpreting findings. The psychoeducational program might have provided the participants with understandings and a language to reflect on such experiences. It is also possible that those who were unwilling to participate in this study faced more challenges in their backgrounds and had less success in coping with their life situations, such that their stories might have showcased fewer resilience factors. Therefore, our findings may highlight the resilience perspective more prominently than if this study had included those who chose not to participate. The coping and resilience perspective also represents a significant shift for the research team. The researchers have experience working in the field of SUD treatment, and we recognize that our initial preconceptions were primarily focused on risk factors and negative outcomes for children and youth growing up in families affected by substance use. Based on the coping strategies employed by the participants, we engaged in on-going discussions to challenge and revise our perspectives during the project. As we gained new insights, our mindset shifted towards a more salutogenic perspective, becoming more aware of factors that promote well-being and coping in this population.

## Conclusion

Children and youth growing up with substance use in the family experience a life course marked by adversity. At the core of their burden, we found the “double-edged sword” of keeping the family secret of the substance use. Although some of their coping actions are necessary, the strains to their mental health may be potentially problematic in the long term. However, the participants also skilfully applied coping strategies that aimed to foster independence and focus on constructing a better and healthy future for themselves. Discussing these efforts to cope in the light of SMH, and especially the construct of SOC, provides us with deeper insight into this complex expression and balance of adversities and capabilities. Furthermore, SOC provides valuable directions for practitioners regarding the domains in which to exert effort in strengthening resilience and mediating the movement towards better health for children and youth affected by substance use. The findings have relevance for the development of intervention strategies and psychoeducational initiatives, underscoring the usefulness of SOC as a framework for systematically identifying and mapping potential resistance resources.

## Data Availability

Data supporting this qualitative study cannot be made available due to ethical considerations, safeguarding participants confidentiality.
